# Coping Patterns over Time and the Association with Stress, Depression and Self-Efficacy Among Adolescents: Latent Transition Analysis

**DOI:** 10.3390/children13010118

**Published:** 2026-01-13

**Authors:** Hye Jeong Choi, Yu Lu, Vi Donna Le, Jeff R. Temple

**Affiliations:** 1 Department of Health Sciences, University of Missouri, Columbia, MO 65211, USA; 2 Department of Health and Exercise Science, University of Oklahoma, 1401 Asp Ave., Norman, OK 73019, USA; yu.lu@ou.edu; 3 Independent Researcher, Roswell, GA 30075, USA; 4 School of Behavioral Health Sciences, University of Texas Health Houston, Houston, TX 77030, USA; jeffrey.r.temple@uth.tmc.edu

**Keywords:** latent transition analysis, coping, transition, gender, adolescents, depressive symptoms, stress, self-efficacy

## Abstract

Introduction: Middle adolescence involves increasingly complex stressors, yet it remains unclear how coping strategies cluster into distinct profiles, how those profiles change across time, and whether profile structure is comparable across gender. We used latent class and transition analysis across three annual waves to identify coping profiles, model transitions, and examine perceived stress, depressive symptoms, and general self-efficacy by profile. Methods: Participants were 964 adolescents (mean age = 16.1 years; 56% female) from public high schools in Texas who completed surveys in spring 2011 with two annual follow-ups. The sample self-identified as Hispanic (32%), White (30%), African American (27%), or other (11%). Latent class/transition models estimated profile membership, transitions, and gender differences in prevalence and transition probabilities. Results: Four coping profiles emerged: Minimal Copers, Maximum Copers, Introverted Approach–Avoidant Copers, and Independent Problem-Solving Copers. Profile structure was comparable for females and males, although prevalence and transition differed. At Wave 4, Introverted Approach–Avoidant Copers reported the highest perceived stress and depressive symptoms, whereas Minimal and Independent Problem-Solving Copers reported lower perceived stress and depressive symptoms. Independent Problem-Solving and Maximum Copers reported higher general self-efficacy, whereas Minimal Copers reported the lowest. Conclusions: Coping in adolescence is heterogeneous and shifts over time, with gender differences in profile prevalence and transitions; findings highlight potential targets for tailored support and self-efficacy enhancement.

## 1. Introduction

Adolescence is a developmental period characterized by substantial biological, cognitive, and social changes [1,2,3]. These changes often elevate stress levels, which can significantly influence psychosocial health [4]. Coping refers to the emotional, cognitive, and behavioral processes individuals use to manage stressful experiences [5]. Transactional models of stress and coping emphasize that coping reflects ongoing, context-sensitive efforts shaped by cognitive appraisals and perceived resources and thus can vary across time and situations [6,7]. Two primary frameworks for modeling coping include dispositional coping, which reflects habitual coping tendencies across situations, and situational coping, which refers to responses to specific stressors [8]. The present study focuses on dispositional coping and its development during middle adolescence. Given the complexity of coping and individual differences in acquiring coping skills [5,9], this study examines longitudinal changes in dispositional coping patterns.

Adolescents typically employ multiple coping strategies, including seeking social support, problem-solving, emotional venting, and distraction [10,11]. However, the field lacks consensus on how to operationalize coping, including measurement methods (e.g., observation vs. self-report), instrumentation, and analytic approaches, thereby limiting a comprehensive understanding [5]. Compas and colleagues [5] have criticized prior research for emphasizing aggregate coping scores while neglecting individual and contextual variability and the multidimensional nature of coping. Similarly, reliance on broad binary dimensions (e.g., problem-focused vs. emotion-focused coping [7]) may obscure the combined effects of multiple strategies. A major review of coping classification systems argues that commonly used higher-order dichotomies (e.g., problem-focused coping vs. emotion-focused) are conceptually problematic because they can aggregate functionally heterogeneous strategies within the same category, and it recommends alternative classification approaches that prioritize functionally homogeneous coping families or action types [12]. Such conceptual measurement issues may help explain why associations between broad coping dimensions and psychological outcomes can appear inconsistent across studies [12]. Although problem-focused coping is often associated with better psychological outcomes, its relationship with distress is inconsistent [13]. Ignoring emotional responses to stressors, such as sadness, can undermine psychological well-being [14]. Bonanno and Burton [15] cautioned against assuming the uniform adaptiveness of any single coping strategy and instead highlighted the importance of flexible strategy deployment in response to situational demands. Consistent with this perspective, a meta-analytic review [16] reported that coping flexibility is positively associated with psychological adjustment. Accordingly, adaptive coping may depend less on any single strategy than on the capacity to match and shift among coping responses as situational demands change [15,16].

To address these limitations, a person-centered approach that identifies subgroups of individuals with similar patterns of coping strategy use across multiple strategies offers a more nuanced understanding than variable-centered approaches that examine coping dimensions independently. Although prior studies have explored coping subgroups [10], many have focused on younger adolescents [11] or specific populations [10], leaving developmental change in coping subgroups less well understood. Variable-centered analyses summarize how coping strategies relate to outcomes at the sample level. When the population is heterogeneous, such averages may fail to represent any particular segment of individuals and may obscure qualitatively different coping profiles and profile-specific coping-outcome relations [17,18]. In contrast, person-centered mixture models use individuals’ multivariate response patterns across coping indicators to identify latent coping profiles, thereby recovering configurations that can be masked by sample-average associations [19,20]. Empirical adolescent studies [10,21,22] using latent profiles/class approaches have repeatedly identified multiple coping profiles and shown that these profiles differentiate internalizing symptoms such as depression and anxiety, supporting the value of examining coping configurations rather than single strategies. Conceptually, coping profiles represent distinct configurations of coping efforts that may show developmental change across adolescence. Within this framework, general self-efficacy is conceptualized as a personal resource relevant to coping strategy selection and use, whereas perceived stress and depressive symptoms are treated as distress-related indicators of psychosocial adjustment that may differ across coping profiles. For clarity, we describe these latent subgroups substantively as coping profiles; analytically, they are estimated as latent classes in the wave-specific latent class analysis and latent statuses in the latent transition analysis.

Developmental-ecological theory emphasizes the importance of context and developmental timing in understanding behavioral tendencies [23,24]. From middle to late adolescence, cognitive growth enables youth to manage increasingly complex stressors [25]. Middle adolescents often face career-related and social challenges and expand their peer networks. Older adolescents tend to use a broader range of coping strategies than younger adolescents [24]. This flexibility is partly attributable to advances in metacognition and executive functioning [25,26]. Prior research [27] has primarily examined coping responses to specific stressors, with limited attention to developmental variation. More longitudinal studies are needed to explore how coping patterns evolve from mid- to late adolescence.

Gender differences in adolescent coping have been widely studied, but findings remain inconsistent. Generally, female adolescents seek social support more frequently than males [27,28,29,30]. Matud [31] reported that females were more likely to use emotional and avoidance coping and less likely to employ rational and detachment strategies. Eschenbeck et al. [27] found that girls used more social support and problem-solving strategies, whereas boys relied more on avoidance. While some studies report similar trends for avoidance coping [32], others suggest boys are more avoidant [33]. Notably, most prior research has examined gender differences in individual coping strategies rather than multidimensional coping profiles and rarely considered developmental stage.

General self-efficacy captures a generalized confidence that one can handle unfamiliar tasks and manage different stressors effectively [34]. In social cognitive theory [35], efficacy beliefs shape whether individuals initiate coping behavior, how vigorously they pursue it, and how long they persist when difficulties arise. Efficacy expectations function as an appraisal-related resource that informs how people evaluate coping options and respond to demanding situations [36]. Empirically, among Italian adolescents, higher general self-efficacy predicted greater use of problem-solving and emotional regulation strategies when managing minor stressors [37]. Thus, general self-efficacy likely varies across coping profiles. Research on resilience further supports this notion, showing differences in perceived self-efficacy between resilient and non-resilient individuals [38].

The role of coping in stress adjustment is well established [38]. Perceived stress levels influence adolescents’ coping choices; for example, youth experiencing high or uncontrollable stressors are more likely to adopt avoidant strategies [11,39]. Conversely, coping can also affect stress outcomes. Active coping generally reduces stress, whereas avoidant coping is associated with heightened stress [40].

Depression, a psychological outcome strongly correlated with stress [41], is shaped by coping effectiveness. Adaptive coping can mitigate depressive symptoms, while maladaptive coping exacerbates them [42]. Avoidant or disengagement coping is consistently linked to higher depression levels [10,43]. Adolescents who use a broader range of coping strategies, even at lower or higher frequencies, report fewer depressive symptoms compared to those relying primarily on avoidance [10]. These findings underscore that coping patterns are closely tied to variations in stress and depression.

Using longitudinal data from a large, ethnically diverse sample of adolescents, this study aimed to identify latent coping classes and to examine transitions among latent coping statuses over three years. We also compared coping profiles and transition patterns by gender and explored associations among coping status, general self-efficacy, stress, and depressive symptoms. Although prior research has applied person-centered approaches to adolescent coping [10], only a handful of studies [44] have investigated whether coping patterns change over time and how these transitions occur. We hypothesized that adolescents would cluster into distinct coping classes and that gender differences would emerge in both prevalence and transitions. Finally, we examined whether coping classes were associated with differences in general self-efficacy, perceived stress, and depressive symptoms.

## 2. Materials and Methods

### 2.1. Participants and Study Design

Current data are from Wave 2, 3, and 4 of *Dating It Safe*, a longitudinal study of teen dating violence and other adolescent health behaviors. Relevant items were not measured at Wave 1. The study procedure was approved by the institutional review board at the University of Texas Medical Branch and informed consent was obtained from all participants included in the study. Nine hundred sixty-four adolescents (Mage = 16.1 years, SD = 0.79) in southeast Texas participated in the survey at Wave 2 (retention rate based on Wave 1: 93%). with annual follow-up assessments. Retention rates based on Wave 1 were 85.8% at Wave 3 and 74.5% at Wave 4, respectively. Participants were 56% female and self-reported as Hispanic (32%), White (30%), African American (27%), and Other (11%). Most participants at Wave 2 were 10th (73%) or 11th (24%) graders. Participants reported living with both parents (48%), mother only (23%), one parent and one-step-parent (20%), father only (3%), other arrangements (3%), and grandparents (3%). Participants were recruited from mandatory classes at seven public high schools in southeast Texas and were not selected based on teen dating violence experiences. The majority of participants were surveyed during school hours at the high school they were attending. Students who graduated or were no longer attending their original high school completed the survey at a convenient location or via an online survey. Participants were reimbursed with $10 gift cards as compensation at Waves 2 and 3, and $20 at Wave 4. At Wave 4, a majority of participants were 12th graders (73%), and the rest were first-year college students (9%), second-year college students (9%), and working or in other situations (9%). Regarding parental socioeconomic status, 13% reported being poor, 68% reported being average, and 19% reported being “pretty well off”.

### 2.2. Measurement

Coping strategies (Latent class/transition analysis indicators) were assessed using the KidCope scale [45], which includes eight items, each representing a distinct coping strategy (see Table 1).

KidCope is a widely used self-report measure of coping in adolescents ages 13 years and up [45]. This scale was used to assess adolescent coping with a wide range of stressful situations and events (e.g., hurricanes: [46]). Prior psychometric evaluations provide evidence supporting the construct validity of the KidCope [46] and reliability evidence (e.g., short-interval test–retest stability) was reported in the original scale development work [45]. Participants received the following instructions: “Now we want to find out how people deal with different problems. Think about a situation that has bothered you during the LAST MONTH and answer the following questions. The problem situation can be about anything (for example, with friends, family, parents, school, or anything else)”. Participants reported how often, “I thought about something else or tried to forget it (distraction)”, “I just handled the situation on my own (social withdrawal)”, “I tried to see the good side of things (cognitive reconstructing)”, I thought of ways to actually solve the problem (problem solving), “I talked about how I was feeling; yelling, screamed, or hit something (negative emotional regulation)”, “I tried to calm myself by talking to myself, praying, taking a walk, or just trying to relax (positive emotional regulation)”, “I kept thinking and wishing this had never happened (wishful thinking)”, “I turned to my family, friends, and other adults to help me feel better (social support)”. We retained the standard KidCope instruction to select a stressor from the past month [45], because this reference period anchors coping reports to a recent, salient event while reducing the likelihood of long-term recall error. The internal consistencies were 0.64~0.65. Negative and positive emotional regulation are two items measuring the same strategy—emotional regulation. Since the two ways of coping are very different (i.e., externalizing versus internalizing behavior) and can be meaningful in identifying different coping profiles, they were considered as two different strategies in the present study. Participants reported their frequency of using each coping strategy during the past month on a four-point scale (1 = not at all and 4 = almost all the time). We created eight binary variables representing youth who infrequently used a specific coping strategy (i.e., combining “not at all” and “sometimes” responses) and youth who frequently used the same specific coping strategy (i.e., combining “a lot of the time” and “almost all the time” responses).

General Self-efficacy (W4) Ten items measured general self-efficacy [47] on a five-point scale (1 = not at all true and 5 = exactly true). We averaged item scores indicating that higher scores had higher general self-efficacy (M = 3.04, SD = 0.56, Internal consistency = 0.92). Example items included: “If I am in trouble, I can usually think of a solution” and “I can usually handle whatever comes my way”.

Perceived stress (W4) Six items were adopted from a perceived global stress scale [48]. Participants reported their perceived stress level by answering the six items on a five-point scale (1 = never and 5 = very often). Example items included: “Been upset because of something that happened unexpectedly?”; “Felt that you were unable to control the important things in your life?”. We averaged items (M = 2.68, SD = 0.95, Internal consistency = 0.90) with higher scores indicating higher levels of perceived stress.

Depressive Symptoms (W4) Eight items were adopted from the Center for Epidemiologic Studies’ Short Depression Scale [49]. Youth reported their past-week depression scores by answering the items on a four-point scale (1 = less than 1 day and 4 = 5–7 days). Example items included: “I had trouble keeping my mind on what I was doing”; “I felt depressed”. Higher scores indicated higher depressive symptoms (M = 1.81, SD = 0.61, Internal consistency = 0.81).

### 2.3. Data Analysis

Latent class analysis (LCA) and transition analysis were conducted in Mplus 7.11 [50] to identify different subgroups of coping strategies and to examine changes in subgroup membership over time [51]. Latent transition analysis (LTA) models longitudinal changes in an unobserved categorical variable (latent status) across multiple measurement occasions using (1) a measurement component defined by class-/status-specific item-response probabilities and (2) a structural component defined by status prevalences and transition probabilities [51]. For clarity, we refer to the latent subgroups as “classes” in the cross-sectional latent class analysis and “statuses” in the latent transition analysis.

#### 2.3.1. Step 1: Latent Class Enumeration at Each Wave

We first estimated latent class models separately at each wave to identify the number of coping classes. Models were estimated beginning with a one-class solution, and additional classes were added sequentially until fit no longer improved. Class enumeration was guided by multiple criteria: (1) (Adjusted) Bayesian information criterion (smaller values indicate better fit), (2) the adjusted Lo–Mendell–Rubin likelihood ratio test (LMRT, [52]), where *p* < 0.05 indicates a significant improvement in fit of the k-class model relative to the k-1 class model [53], and (3) theoretical interpretation [53]. Because likelihood-ratio tests (e.g., Lo–Mendell–Rubin likelihood ratio test) and information criteria can occasionally yield different recommendation in mixture models, we applied a multi-criteria decision rule, prioritizing Bayesian information criterion and sample-size adjusted Bayesian information criterion alongside Lo–Mendell–Rubin likelihood ratio test and interpretability rather than considering Lo–Mendell–Rubin likelihood ratio test as the sole decision criteria [53]. Across waves, a four-class solution was selected (See Table 2). At Wave 2, the distinctiveness of the fourth class was relatively weaker (i.e., non-significant LMRT and negligible difference in unadjusted BIC relative to the 3-class model), but the four-class solution was retained to support longitudinal consistency and interpretability across waves in the latent transition analysis.

#### 2.3.2. Step 2: Latent Transition Analysis and Longitudinal Measurement Invariance

After identifying the number of classes at each wave, we estimated an LTA model across Waves 2–4 to examine transitions between coping statuses over time. We evaluated longitudinal measurement invariance by comparing (1) an LTA model that constrained the class-specific item-response probabilities to be equivalent across waves (BIC = 25,415.73) and (2) a model that allowed these probabilities to vary across waves (BIC = 33,161.61). This approach aligns with standard LTA practice for determining whether the same latent statuses are measured equivalently over time [51]. The invariant model showed better fit (lower BIC), supporting measurement invariance across waves (see Table 3). Accordingly, the invariant measurement model was retained, indicating that the same coping statuses were measured equivalently over time.

#### 2.3.3. Step 3: Gender Invariance and Moderated Transitions

To test whether coping classes were comparable for females and males, we separately conducted female and male latent class analysis models at each wave and compared models that constrained item-response probabilities to be equivalent across gender versus models that allowed these probabilities to differ. Across all waves and gender, four classes were identified (See Table 2). Gender invariance was evaluated by comparing constrained versus unconstrained models at Wave 2 (BIC = 11,046.85 vs. 11,136.33), Wave 3 (BIC = 10,195.95 vs. 10,318.21), and at Wave 4 (BIC = 8443.40 vs. BIC = 8567.97). We also compared two latent transition analysis models: (1) a model with longitudinal measurement invariance and gender invariance (BIC = 27,108.33) and (2) a model allowing item-response probabilities to vary across gender over time (BIC = 27,174.71). Because the invariant models consistently showed lower BIC, we concluded that the four-class structure was comparable across females and males over time (See Table 3). As noted in Step 1, evidence for the fourth class at Wave 2 was relatively weaker (i.e., a non-significant LMRT and a negligible difference in unadjusted BIC relative to the 3-class model; Table 2). Nevertheless, the four-class structure was retained for longitudinal consistency in the LTA. Therefore, rather than estimating separate LTAs for females and males, we fit the latent transition analysis in the full sample and examined gender differences by estimating gender-specific prevalences and transition probabilities by adding gender as a moderator.

#### 2.3.4. Missing Data, Covariates, and Distal Outcomes

Missingness was dealt with using a full information maximum likelihood (FIML) method [54]. We also included ethnicity, parental education, and age in the LCA as covariates and tested whether these variables predicted membership in coping classes at Wave 2. None of the variables predicted membership in classes at *p* < 0.05, and they were not retained in subsequent models. Finally, using a 3-step latent class analysis with BCH procedure [55], we examined associations between Wave 4 coping status and Wave 4 outcomes such as general self-efficacy, perceived stress, and depressive symptoms.

## 3. Results

Table 1 presents the proportion of adolescents who endorsed the frequent-use category for each coping strategy at each wave. At Wave 2, the highest proportions of frequent endorsement were observed for social withdrawal, cognitive reconstructing, problem solving, and positive emotional regulation, whereas the lowest proportions were observed for distraction, negative emotional regulation, and wishful thinking. These descriptive item-level frequencies summarize endorsement of each strategy in the overall sample and do not represent latent coping statuses, which are defined by multivariate patterns of strategy endorsement (Section 3.1 and Section 3.2).

### 3.1. Latent Coping Status

Four coping statuses (i.e., classes/subgroups) were identified across Waves 2–4 (see Table 2). Status labels were assigned based on the status-specific item-response probabilities, which represent the probability of endorsing the frequent-use category for each coping strategy conditional on status membership (see Table 4). To facilitate interpretation, we summarize each status by its most and least likely strategies.

1. Minimum Coping. Low probabilities of frequent endorsement across all eight coping strategies. (*Prototype:* unlikely to endorse frequent use of any strategy.)
2. Maximum Coping. High probabilities of frequent endorsement for most strategies, with a moderate probability of negative emotion regulation. (*Prototype:* likely to endorse frequent use of many strategies, including both active and avoidant/emotion-focused strategies.)
3. Introverted Approach-Avoidant Coping. Highest probability of frequent wishful thinking; moderate probabilities of distraction, social withdrawal, and positive emotion regulation; comparatively lower probabilities of frequent problem solving and social support. (*Prototype:* likely to endorse wishful thinking and more private/avoidant strategies, and less likely to endorse active/social approaches.)
4. Independent Problem Solving. High probabilities of frequent social withdrawal, cognitive reconstructing, problem solving, and positive emotion regulation; moderate probability of social support; low probabilities of frequent distraction, negative emotion regulation, and wishful thinking. (*Prototype:* likely to endorse cognitive/problem-solving and self-regulation strategies, unlikely to endorse wishful thinking or negative emotion regulation.)

The Independent Problem Solving status was the most prevalent status overall and increased across Waves 2–4. The Minimal Coping status was least prevalent and decreased over time. The Introverted Approach–Avoidant status decreased across waves, whereas the Maximum Coping status increased and became the second most prevalent status by Wave 4.

### 3.2. Transition Between Latent Coping Statuses

The pooled LTA model (i.e., not including gender as a moderator) indicated that adolescents generally remained in the same status across adjacent waves (See Table 5). Table 5 presents the transition probability matrix (rows = Wave t status; columns = Wave t + 1 status), where diagonal values indicate stability and off-diagonal values indicate transitions. When transitions occurred, movements most frequently shifted toward Independent Problem Solving, although the magnitude and direction of off-diagonal transitions differed by starting status and wave. 

From Wave 2 to Wave 3. Stability was the dominant pattern across all statuses. Among adolescents who transitioned, those in Minimal Coping and Maximum Coping most often transitioned into Independent Problem Solving (and, to a lesser extent, Introverted Approach–Avoidant). Adolescents in Independent Problem Solving who transitioned most often moved into Maximum Coping. The Introverted Approach–Avoidant status showed more diffuse transitions across the other three statuses.

From Wave 3 to Wave 4. Stability remained the most common pattern. For adolescents who transitioned out of Minimal Coping or Maximum Coping, transitions most frequently moved into Independent Problem Solving. As in the prior interval, transitions out of Independent Problem Solving most commonly moved toward Maximum Coping, whereas transitions from Introverted Approach–Avoidant were distributed across the other statuses. 

#### Prevalence of Coping Status, and Transition in Gender

Although females and males had similar coping patterns across time (see Table 2), the prevalence of each status and transition probabilities varied (see Table 4 and Table 5).

*Girls*. At Wave 2, the most prevalent status was Introverted Approach–Avoidant (38.6%), which declined across waves (Wave 4: 23.0%). Maximum Coping increased from 27.5% (Wave 2) to 37.7% (Wave 4). Minimal Coping remained the least prevalent status but increased from 7.5% (Wave 2) to 12.6% (Wave 4). Prevalence of Independent Problem Solving was relatively stable across waves (Table 3). Transition patterns in females were broadly similar to the pooled model, with high stability across waves. Notably, when females transitioned out of Minimal Coping, transitions most often moved to Introverted Approach–Avoidant or Independent Problem Solving, and transitions out of Introverted Approach–Avoidant most often moved toward Maximum Coping.

**Table 4:** Prevalence and Item-Response Probabilities in Latent Coping Strategy Status.

	Minimum Coping	Maximum Coping	Introverted Approach-Avoidance Coping	Independent Problem Solving
Prevalence of Statuses (%)				
Wave 2				
Total sample	152 (15.46)	217 (22.07)	287 (29.20)	327 (33.27)
Female (*n* = 549)	41 (7.47)	151 (27.50)	212 (38.62)	145 (26.41)
Male (*n* = 434)	111 (25.58)	66 (15.21)	75 (17.28)	182 (41.93)
Wave 3				
Total sample	119 (12.11)	237 (24.11)	248 (25.23)	379 (38.55)
Female (*n* = 549)	54 (9.84)	166 (30.24)	179 (32.60)	150 (27.32)
Male (*n* = 434)	65 (14.98)	71 (16.36)	69 (15.90)	229 (52.76)
Wave 4				
Total sample	119 (12.11)	291 (29.60)	171 (17.39)	402 (40.90)
Female (*n* = 549)	69 (12.57)	207 (37.70)	126 (22.95)	147 (26.78)
Male (*n* = 434)	50 (11.52)	84 (19.35)	45 (10.37)	255 (58.76)
Item-response probabilities				
Distraction				
Infrequent	**0.938**	0.279	0.419	**0.808**
Frequent	0.062	**0.721**	**0.581**	0.192
Social Withdrawal				
Infrequent	**0.763**	0.257	0.433	0.278
Frequent	0.237	**0.743**	**0.567**	**0.722**
Cognitive reconstructing				
Infrequent	**0.692**	0.153	**0.614**	0.171
Frequent	0.308	**0.847**	0.386	**0.829**
Problem solving				
Infrequent	**0.837**	0.193	**0.657**	0.137
Frequent	0.163	**0.807**	0.343	**0.863**
Negative emotional regulation				
Infrequent	**0.997**	**0.593**	**0.729**	**0.922**
Frequent	0.003	0.407	0.271	0.078
Positive emotional regulation				
Infrequent	**0.918**	0.063	0.484	0.398
Frequent	0.082	**0.937**	**0.516**	**0.602**
Wishful thinking				
Infrequent	**0.968**	0.180	0.304	**0.837**
Frequent	0.032	**0.820**	**0.696**	0.163
Social support				
Infrequent	**0.907**	0.348	**0.727**	**0.525**
Frequent	0.093	**0.652**	0.273	0.475

Note: Boldface numbers represent moderate to high probabilities.

**Table 5:** Transition Probabilities in Latent Coping Strategy Status by Gender across Times.

	Pooled *				Girls				Boys			
**W2 to W3**	Minimal	Maximum	Introverted Approach-Avoidant	Independent Problem Solving	Minimal	Maximum	Introverted Approach-Avoidant	Independent Problem Solving	Minimal	Maximum	Introverted Approach-Avoidant	Independent Problem Solving
Minimal	**0.443**	0.089	0.187	0.281	**0.496**	0.140	0.196	0.169	**0.421**	0.063	0.183	0.328
Maximum	0.045	**0.769**	0.083	0.103	0.016	**0.769**	0.096	0.119	0.109	**0.768**	0.054	0.070
Introverted Approach-Avoidant	0.104	0.097	**0.691**	0.108	0.119	0.138	**0.699**	0.044	0.067	0.000	**0.672**	0.261
Independent Problem Solving	0.046	0.099	0.051	**0.804**	0.024	0.127	0.111	**0.738**	0.065	0.074	0.000	**0.861**
**W3 to W4**	Minimal	Maximum	Introverted Approach-Avoidant	Independent Problem Solving	Minimal	Maximum	Introverted Approach-Avoidant	Independent Problem Solving	Minimal	Maximum	Introverted Approach-Avoidant	Independent Problem Solving
Minimal	**0.450**	0.108	0.105	0.338	**0.532**	0.035	0.242	0.192	**0.387**	0.164	0.000	0.449
Maximum	0.052	**0.742**	0.063	0.143	0.029	**0.791**	0.090	0.090	0.106	**0.629**	0.000	0.265
Introverted Approach-Avoidant	0.182	0.166	**0.516**	0.136	0.161	0.229	**0.492**	0.118	0.229	0.025	**0.568**	0.178
Independent Problem Solving	0.019	0.215	0.000	**0.766**	0.000	0.301	0.000	**0.699**	0.032	0.157	0.000	**0.811**

Note: * Pooled transition probabilities are based on the LTA model without gender as a moderator.

*Boys*. At Wave 2, the most prevalent status was Independent Problem Solving, which increased over time, whereas Minimal Coping decreased from 25.6% (Wave 2) to 12.0% (Wave 4). Maximum Coping increased from 15.2% (Wave 2) to 19.4% (Wave 4), and Introverted Approach–Avoidant decreased from 17.3% (Wave 2) to 10.4% (Wave 4) (Table 3). Transition patterns in males were also characterized by high stability (Table 4). Relative to the pooled estimates, a notable pattern was that transitions from Maximum Coping were more likely to move toward Minimal Coping in males, whereas transitions from Maximum Coping in the pooled model more often moved toward Independent Problem Solving.

### 3.3. Coping Status and Wave 4 Outcomes

**Figure 1:**
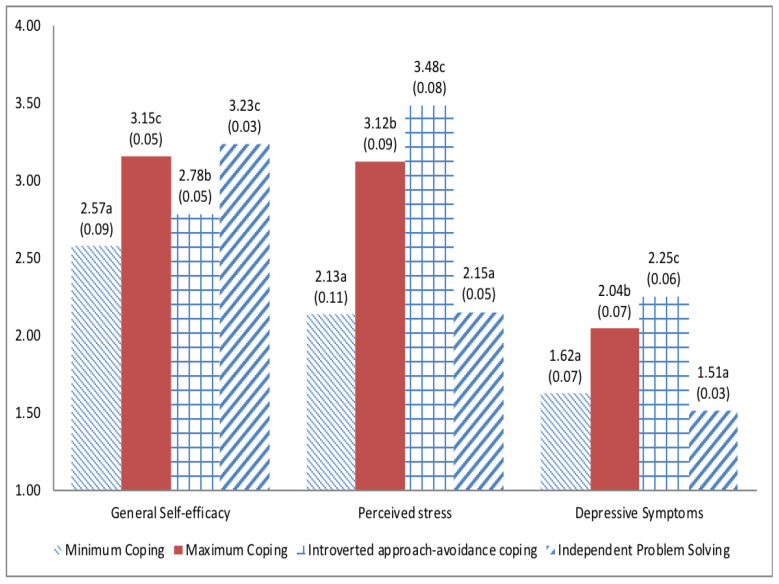
Coping Status Differences in Wave 4 Outcomes.

Given that coping statuses were identified equivalently across waves and gender, we examined differences in Wave 4 outcomes by Wave 4 coping status in the pooled sample. Because coping status and outcomes were assessed at the same wave, these findings represent cross-sectional differences across coping statuses. As shown in Figure 1, adolescents in the Independent Problem Solving and Maximum Coping statuses showed the highest levels of general self-efficacy, whereas the Minimal Coping status showed the lowest levels. In addition, adolescents in the Minimal Coping and Independent Problem Solving statuses reported lower perceived stress than those in the Introverted Approach–Avoidant and Maximum Coping statuses. Finally, depressive symptoms were lowest in the Independent Problem Solving and Minimal Coping statuses and highest in the Maximum Coping and Introverted Approach–Avoidant statuses.

Note: Estimates that share any letter are not significantly different at α = 0.05.

## 4. Discussion

Using a large, ethnically diverse sample of adolescents, we identified four latent coping statuses and examined how adolescents transitioned between these statuses across three annual waves; we also compared Wave 4 general self-efficacy, perceived stress, and depressive symptoms across statuses. Prior person-centered studies of adolescent coping have often been cross-sectional and have identified profiles such as low/inactive coping, avoidant coping, and high/active coping, with meaningful differences in internalizing symptoms across these profiles [10,21,22]. Similar broadly elevated patterns have been described in person-centered studies under related labels (e.g., high/active or globally engaged coping profiles), suggesting that our ‘Maximum Coping’ status aligns with a previously observed profile under different nomenclature [10,21,22]. Extending this literature, the present study applied latent transition analysis to evaluate both the stability and change in coping-status membership across three annual waves and to examine whether the same coping-status structure was comparable across gender. This longitudinal, status-based approach strengthens interpretation of developmental change because it evaluates change in latent status membership rather than only mean-level associations among single coping strategies.

The Maximum Coping status, characterized by frequent endorsement of a broad set of coping strategies, resembles the “Active Copers” profiles reported in prior person-centered studies [21]. In that study [21], “Active Copers” were characterized by above-average endorsement of all coping strategies and reported elevated internalizing symptoms, suggesting that broadly high coping does not necessarily indicate better adjustment and may reflect heightened coping demands or less selective, “try-everything” responding. Consistent with this interpretation [21], youth in the Maximum Coping status in the present study also reported relatively high perceived stress and general self-efficacy (see Figure 1), which may reflect drawing on a wide repertoire of strategies including disengagement/avoidance when stress is elevated [56]. Although such responses can be functional for managing overwhelming or uncontrollable demands in the moment [56], meta-analytic evidence links greater disengagement/avoidance with poorer psychosocial adjustment, including higher internalizing symptoms [5]. Together, these findings underscore that coping adaptiveness is context-dependent and may shift as stressors and available resources change [15,21,56]. Moreover, research on regulatory/coping flexibility [15] cautions against assuming that any single strategy is uniformly adaptive; instead, coping effectiveness depends on how well the strategy fits situational demands and on whether individuals adjust their responses based on feedback over time.

Independent Problem-solving copers engaged in multiple potentially adaptive strategies such as problem-solving and cognitive-reconstructing, which were coping strategies identified to help reduce stress and depression [57]. Independent Problem-Solving copers had the lowest levels of perceived stress and depressive symptoms and the highest general self-efficacy among the four statuses. Adolescents in this status tended to have higher general self-efficacy at Wave 4. Yet, the direction of association cannot be determined because coping status and outcomes were assessed concurrently at Wave 4.

Across adjacent waves, youth in the Maximum and Independent Problem Solving coping statuses were most likely to remain stable. When transitions occurred, they mostly shifted between these two statuses (Maximum ↔ Independent Problem Solving). Consistent with the overall transition patterns, transitions out of Minimal Coping and Maximum Coping also most often shifted toward Independent Problem Solving, suggesting a broader developmental trend toward more problem-solving/regulation-focused configurations. Notably, both coping statuses included multiple (typically) adaptive strategies such as problem-solving and cognitive-reconstructing. Because adolescents ages 15–20 showed increases in positive problem-solving [24], youth in both groups may become increasingly able to select strategies in ways that fit situational demands.

Minimal copers engaged less frequently in all coping strategies compared with the other three statuses and exhibited lower levels of general self-efficacy, perceived stress, and depression. A certain portion of adolescents are less likely to engage in all types of coping strategies [10,44]. This profile is theoretically unidentified but is continuously found empirically [10,44]. We observed a decreased prevalence of Minimal Coping over time and this status showed comparatively lower stability and greater movement into other statuses. Because we did not directly assess life-event exposure or personality, these possibilities should be considered hypotheses for future research rather than conclusions. Future studies should test whether low-coping patterns reflect fewer coping demands, different stress appraisal, or other individual resources (e.g., personality) measured directly. Studies [58] suggest that personality traits can relate to perceived stress and coping. However, testing these mechanisms will require direct measurement of personality and life-event exposure. Although Minimal Coping was characterized by lower distress in the present data, the combination of low strategy endorsement and lower general self-efficacy may indicate fewer coping resources if stressors intensify; this possibility warrants future study. Thus, universal prevention efforts that strengthen coping skills may still be beneficial, including for adolescents who report low coping engagement.

Introverted Approach-Avoidant copers had the highest stress and depression levels albeit a relatively low level of general self-efficacy. Given that their higher utilization of avoidance coping strategies (e.g., wishful thinking) and lower employment of active coping strategies (e.g., problem-solving), they may be less able to manage their higher level of stress [5]. While youth in the Introverted Coping status were most likely to remain in that status over time, a sizeable number moved to other coping statuses. Although the exact mechanism is unknown, some coping strategies (e.g., wishful thinking) that characterize the Introverted Coping status tend to decrease in adolescence [24]. Youth in this status may perceive their coping efforts as less effective, and some may lack the resources, support, or skills to adopt alternative strategies. Our study findings highlight the need for identifying and targeting this group of adolescents to better manage stressful situations.

Taken together, the Wave 4 outcome pattern suggests that high endorsement of many coping strategies (Maximum Coping) can co-occur with elevated distress, whereas a more selective configuration emphasizing problem solving/cognitive restructuring and regulation (Independent Problem Solving) aligns with the lowest distress.

Overall, males and females showed the same set of coping statuses across waves, with gender differences expressed primarily in prevalence and transition probabilities. In the present study, measurement invariance testing indicated that the same set of coping statuses could be meaningfully interpreted for females and males, suggesting that gender differences were expressed primarily in prevalence (and transition probabilities) rather than in qualitatively different coping-status structures. Consistent with prior person-centered work [21], coping profiles can be similar across gender while their distributions differ. For example, “Inactive Copers” tend to include a higher proportion of boys in some samples [21]. Practically, this suggests that interventions may not require different coping-taxonomies by gender, but may benefit from gender-informed targeting toward the statuses that are more prevalent or more persistent within each gender. For female adolescents, the finding that Introverted Approach-avoidance Coping status was the most prevalent coping status at Wave 2 was somewhat consistent with Matud’s [31] results where females were more likely to use emotional and avoidance coping styles than other coping styles. The prevalence of Introverted Approach-Avoidance Coping status decreased over time; Maximum Coping became the most prevalent status among females by Wave 4. This pattern may reflect increasing endorsement of a broader coping repertoire over time (i.e., greater representation in the Maximum Coping status) and reduce likelihood of endorsing less active coping strategies (e.g., wishful thinking). For male adolescents, problem solving seemed to be a frequently endorsed strategy, consistent with the overall transition trend toward Independent Problem Solving. Across the study period, this may reflect greater reliance on strategies that are more consistently linked to better adjustment in prior work, relative to disengagement/avoidance coping [5].

Several limitations should be considered. First, measurement alignment between coping and stress should be noted. Coping was assessed with reference to a self-identified stressful situation during the past month (KidCope), whereas perceived stress reflects a global appraisal of stress-related feelings and thoughts during the past month. Because stressor-anchored coping reports and global perceived stress capture related but non-identical constructs, associations between coping status and perceived stress should be interpreted with this distinction in mind. Moreover, we did not directly measure objective stressor exposure or systematically characterize the type/severity of the self-identified stressor. Future research should examine whether coping-status patterns and transitions differ across stressor contexts. Second, coping strategy coverage and measurement limitations may have influenced the identified statuses. Although several of our coping statuses (e.g., Minimal, Independent Problem Solving, Introverted Approach-Avoidant) conceptually align with coping profiles in previous studies (e.g., Low Generic Copers, Active Copers, and Avoidance Copers in Aldridge and Roesch [10]), not all types of coping strategies (e.g., humor) were included, which may have limited identification of additional coping statuses. Similarly, coping was measured using eight single-item indicators, and internal consistency for an aggregated KidCope score in our sample was modest (α ≈ 0.64–0.65). This is not unexpected for brief checklists that intentionally assess heterogeneous coping strategies (one item per coping category), and Cronbach’s alpha should be interpreted cautiously because it depends on dimensionality and item count [59]. Relatedly, because each coping strategy in the KidCope is assessed with a single item, strategy-level measurement error and limited content coverage may have affected class separation and the precision of status prevalence/transition estimates. A more robust measure may yield different coping statuses and different estimates of status prevalence and transition probabilities. The coping measure was further limited by a reliance on emotional social support and did not include instrumental social support. In addition, dichotomizing the original 4-point response scale may have reduced information and introduced sensitivity to the chosen cut-points. Third, the study did not include specific situational or personal stressors such as parental divorce. Fourth, psychosocial outcomes (perceived stress, depressive symptoms, and general self-efficacy) were assessed only at Wave 4. Therefore, associations between coping status and these outcomes are cross-sectional and do not establish temporal precedence or the impact of coping transitions on outcomes. Fifth, the timing of data collection may limit generalizability to today’s adolescents. Because these data were collected in 2011–2013, changes over the past decade in the sociocultural context of adolescence and the nature of external stressors may limit the direct generalizability of the findings to today’s youth, underscoring the need for replication in more recent cohorts. Finally, our study identified coping statuses among community-based adolescents in mid- to late-adolescence in Texas. Future research should also examine whether coping-status prevalence and transition patterns vary across sociodemographic subgroups (e.g., ethnicity and indicators of socioeconomic context), particularly in samples that support adequately powered subgroup comparisons. These statuses need to be further tested and verified with different adolescent samples across age groups and geographic contexts.

## 5. Conclusions

Our findings underscore the dynamic nature of coping patterns from middle to late adolescence and highlight the value of longitudinal research in understanding these changes. Interventions designed to strengthen coping skills are particularly needed for adolescents in the less adaptive coping statuses. Additionally, gender differences emerged in both the prevalence of coping statuses and their transition patterns. These results emphasize the importance of examining coping as a multidimensional construct—considering patterns of multiple strategies rather than focusing on isolated coping behaviors. Future research should replicate these findings in more recent cohorts and assess psychosocial outcomes across multiple waves to better evaluate temporal associations between coping-status transitions and adjustment.

## Figures and Tables

**Table 1:** Distribution of Coping Strategy Use by Wave.

	Wave 2	Wave 3	Wave 4
Coping strategy items	*n* (%)	*n* (%)	*n* (%)
I thought about something else or tried to forget it. (Distraction)			
Infrequent	584 (60.77)	529 (59.37)	432 (56.18)
Frequent	377 (39.23)	362 (43.62)	337 (43.82)
I just handled the situation on my own. (Social withdrawal)			
Infrequent	389 (40.56)	338 (37.98)	265 (34.51)
Frequent	570 (59.44)	552 (62.02)	503 (65.49)
I tried to see the good side of things (Cognitive reconstructing)			
Infrequent	372 (38.91)	309 (34.95)	235 (30.69)
Frequent	584 (61.9)	575 (65.05)	531 (69.32)
I thought of ways to actually solve the problem. (Problem solving)			
Infrequent	412 (43.01)	340 (38.29)	244 (31.85)
Frequent	546 (56.99)	548 (61.71)	522 (68.15)
I talked about how I was feeling; yelled, screamed, or hit something. (Negative emotional regulation)			
Infrequent	782 (81.46)	698 (78.43)	605 (79.29)
Frequent	178 (18.54)	192 (21.57)	158 (20.71)
Tried to calm myself by talking to myself, praying, taking a walk, or just trying to relax (Positive emotional regulation)			
Infrequent	415 (43.14)	365 (41.06)	282 (36.77)
Frequent	547 (56.86)	524 (58.94)	485 (63.23)
I kept thinking and wishing this had never happened. (Wishful thinking)			
Infrequent	519 (54.06)	487 (54.84)	428 (55.95)
Frequent	441 (45.94)	401 (45.16)	337 (44.05)
Turned to my family, friends, or other adults to help me feel better. (Social support)			
Infrequent	600 (62.37)	527 (59.28)	400 (52.29)
Frequent	362 (37.63)	362 (36.71)	365 (47.71)

**Table 2:** Model Fit Indices Latent Coping Class Analysis across Three Waves.

	Wave 2			Wave 3			Wave 4		
Overall sample	BIC	Adj BIC	LMRT	BIC	Adj BIC	LMRT	BIC	Adj BIC	LMRT
1 class									
2 class	9775.73	9721.74	339.82 ***	9076.22	9022.23	302.22 ***	7567.44	7513.46	382.12 *
3 class	9718.04	9635.46	117.62 *	8972.02	8889.45	162.68 ***	7410.08	7327.51	213.60 ***
4 class	9718.27	9607.11	60.62	8965.20	8854.05	66.86 **	7388.56	7277.42	79.98
5 class	9732.64	9592.90	46.70 ***	8976.61	8836.87	48.94	7399.52	7259.80	48.04
Boys									
1 class									
2 class	4105.79	4051.85	219.40 ***	3844.11	3790.17	142.58 ^†^	3062.93	3009.00	171.06 ***
3 class	4093.76	4011.25	65.30 *	3812.53	3730.03	83.78 *	3024.25	2941.78	89.05 *
4 class	4110.78	3999.72	36.77 *	3830.09	3719.04	35.55 ^†^	3035.55	2924.53	40.01
5 class	4138.52	3998.89	26.30	3856.07	3716.46	27.28	3062.29	2922.72	24.87 *
Girls									
1 class									
2 class	5659.13	5605.17	112.65	5227.44	5173.48	161.60 ***	4472.14	4418.19	239.50 ***
3 class	5642.61	5560.07	71.85 *	5202.85	5120.32	79.09 ***	4420.45	4337.94	104.63 *
4 class	5665.53	5554.43	33.08	5223.75	5112.66	34.40 ^†^	4428.24	4317.17	46.21
5 class	5695.56	5555.89	26.10	5251.16	5111.51	28.00	4454.20	4314.56	28.37 *

Note: (Adj) BIC: (Adjusted) Bayesian information criterion LMRT: Lo–Mendell–Rubin likelihood ratio test * *p* < 0.05, ** *p* < 0.01, *** *p* < 0.001, ^†^ *p* < 0.10.

**Table 3:** Longitudinal Measurement Invariance and Gender Invariance Tests.

Test/Model Comparison	Constrained Model (Invariant)	Unconstrained Model (Variant)	BIC (Constrained)	BIC (Unconstrained)	ΔBIC (Unc − Con)	Model Retained
Longitudinal measurement invariance (LTA; Waves 2–4)	Item-response probabilities constrained equal across Waves 2–4	Item-response probabilities allowed to vary across waves	25,415.73	33,161.61	7745.88	Constrained
Gender measurement invariance (LCA; Wave 2)	Item-response probabilities constrained equal across females and males	Item-response probabilities allowed to differ by gender	11,046.85	11,136.33	89.48	Constrained
Gender measurement invariance (LCA; Wave 3)	Item-response probabilities constrained equal across females and males	Item-response probabilities allowed to differ by gender	10,195.95	10,318.21	122.26	Constrained
Gender measurement invariance (LCA; Wave 4)	Item-response probabilities constrained equal across females and males	Item-response probabilities allowed to differ by gender	8443.40	8567.97	124.57	Constrained
Combined longitudinal + gender invariance (LTA; Waves 2–4)	Item-response probabilities constrained equal across waves and gender	Item-response probabilities allowed to vary across waves and gender	27,108.33	27,174.71	66.38	Constrained

Note: BIC = Bayesian information criterion; lower values indicate better relative fit. ΔBIC is computed as BIC (Unconstrained) − BIC (Constrained); positive values favor the constrained (invariant) model. “Model retained” indicates which model was retained based on BIC comparison.

## Data Availability

The data presented in this study are openly available in https://www.icpsr.umich.edu/sites/icpsr/find-data (accessed on 8 January 2026).

## References

[1] Parke R.D., Ladd G.W. (2016). Family-Peer Relationships: Modes of Linkage.

[2] Blakemore S.-J. (2012). Development of the Social Brain in Adolescence. J. R. Soc. Med..

[3] Waters S., Lester L., Cross D. (2014). How Does Support From Peers Compare with Support From Adults as Students Transition to Secondary School?. J. Adolesc. Health.

[4] Doane L.D., Mineka S., Zinbarg R.E., Craske M., Griffith J.W., Adam E.K. (2013). Are Flatter Diurnal Cortisol Rhythms Associated with Major Depression and Anxiety Disorders in Late Adolescence? The Role of Life Stress and Daily Negative Emotion. Dev. Psychopathol..

[5] Compas B.E., Jaser S.S., Bettis A.H., Watson K.H., Gruhn M.A., Dunbar J.P., Williams E., Thigpen J.C. (2017). Coping, Emotion Regulation, and Psychopathology in Childhood and Adolescence: A Meta-Analysis and Narrative Review. Psychol. Bull..

[6] Folkman S., Lazarus R.S. (1985). If It Changes It Must Be a Process: Study of Emotion and Coping during Three Stages of a College Examination. J. Personal. Soc. Psychol..

[7] Lazarus R.S., Folkman S. (1984). Stress, Appraisal, and Coping.

[8] Bouchard G., Guillemette A., Landry-Léger N. (2004). Situational and Dispositional Coping: An Examination of Their Relation to Personality, Cognitive Appraisals, and Psychological Distress. Eur. J. Pers..

[9] Skinner E.A., Zimmer-Gembeck M.J. (2007). The Development of Coping. Annu. Rev. Psychol..

[10] Aldridge A.A., Roesch S.C. (2008). Developing Coping Typologies of Minority Adolescents: A Latent Profile Analysis. J. Adolesc..

[11] Tolan P.H., Gorman–Smith D., Henry D., Chung K., Hunt M. (2002). The Relation of Patterns of Coping of Inner–City Youth to Psychopathology Symptoms. J. Res. Adolesc..

[12] Skinner E.A., Edge K., Altman J., Sherwood H. (2003). Searching for the Structure of Coping: A Review and Critique of Category Systems for Classifying Ways of Coping. Psychol. Bull..

[13] Zimmer-Gembeck M.J., Skinner E.A., Cicchetti D. (2016). The Development of Coping: Implications for Psychopathology and Resilience. Developmental Psychopathology.

[14] Austenfeld J.L., Stanton A.L. (2004). Coping Through Emotional Approach: A New Look at Emotion, Coping, and Health-Related Outcomes. J. Personal..

[15] Bonanno G.A., Burton C.L. (2013). Regulatory Flexibility: An Individual Differences Perspective on Coping and Emotion Regulation. Perspect. Psychol. Sci..

[16] Cheng C., Lau H.-P.B., Chan M.-P.S. (2014). Coping Flexibility and Psychological Adjustment to Stressful Life Changes: A Meta-Analytic Review. Psychol. Bull..

[17] von Eye A., Anne Bogat G. (2006). Person-Oriented and Variable-Oriented Research: Concepts, Results, and Development. Merrill-Palmer Q..

[18] Bergman L.R., Trost K. (2006). The Person-Oriented Versus the Variable-Oriented Approach: Are They Complementary, Opposites, or Exploring Different Worlds?. Merrill-Palmer Q..

[19] Woo S.E., Hofmans J., Wille B., Tay L. (2024). Person-Centered Modeling: Techniques for Studying Associations Between People Rather than Variables. Annu. Rev. Organ. Psychol. Organ. Behav..

[20] Masyn K.E. (2013). Latent Class Analysis and Finite Mixture Modeling. The Oxford Handbook of Quantitative Methods.

[21] Perzow S.E.D., Bray B.C., Wadsworth M.E., Young J.F., Hankin B.L. (2021). Individual Differences in Adolescent Coping: Comparing a Community Sample and a Low-SES Sample to Understand Coping in Context. J. Youth Adolesc..

[22] Herres J. (2015). Adolescent Coping Profiles Differentiate Reports of Depression and Anxiety Symptoms. J. Affect. Disord..

[23] Tolan P.H., Guerra N.G., Kendall P.C. (1995). A Developmental€cological Perspective on Antisocial Behavior in Children and Adolescents: Toward a Unified Risk and Intervention Framework. J. Consult. Clin. Psychol..

[24] Zimmer-Gembeck M.J., Skinner E.A. (2011). Review: The Development of Coping across Childhood and Adolescence: An Integrative Review and Critique of Research. Int. J. Behav. Dev..

[25] Thompson R.A., Goodman M. (2010). Development of Emotion Regulation: More than Meets the Eye. Emotion Regulation and Psychopathology: A Transdiagnostic Approach to Etiology and Treatment.

[26] Skinner E.A., Zimmer-Gembeck M.J. (2010). Perceived Control and the Development of Coping. Oxford Handbook of Stress, Health, and Coping.

[27] Eschenbeck H., Kohlmann C.-W., Lohaus A. (2007). Gender Differences in Coping Strategies in Children and Adolescents. J. Individ. Differ..

[28] Brittian A.S., Toomey R.B., Gonzales N.A., Dumka L.E. (2013). Perceived Discrimination, Coping Strategies, and Mexican Origin Adolescents’ Internalizing and Externalizing Behaviors: Examining the Moderating Role of Gender and Cultural Orientation. Appl. Dev. Sci..

[29] Flannery K.M., Vannucci A., Ohannessian C.M. (2018). Using Time-Varying Effect Modeling to Examine Age-Varying Gender Differences in Coping Throughout Adolescence and Emerging Adulthood. J. Adolesc. Health.

[30] Piko B. (2001). Gender Differences and Similarities in Adolescents’ Ways of Coping. Psychol. Rec..

[31] Matud M.P. (2004). Gender Differences in Stress and Coping Styles. Personal. Individ. Differ..

[32] Griffith M.A., Dubow E.F., Ippolito M.F. (2000). Developmental and Cross-Situational Differences in Adolescents’ Coping Strategies. J. Youth Adolesc..

[33] Winkler Metzke C., Steinhausen H.-C. (2002). Bewältigungsstrategien im Jugendalter. Z. Entwicklungspsychol. Pädagogische Psychol..

[34] Luszczynska A., Scholz U., Schwarzer R. (2005). The General Self-Efficacy Scale: Multicultural Validation Studies. J. Psychol..

[35] Bandura A. (1986). Social Foundations of Thought and Action: A Social Cognitive Theory.

[36] Karademas E.C., Kalantzi-Azizi A. (2004). The Stress Process, Self-Efficacy Expectations, and Psychological Health. Personal. Individ. Differ..

[37] Cicognani E. (2011). Coping Strategies with Minor Stressors in Adolescence: Relationships with Social Support, Self-Efficacy, and Psychological Well-Being: COPING STRATEGIES WITH MINOR STRESSORS. J. Appl. Soc. Psychol..

[38] Galatzer-Levy I.R., Burton C.L., Bonanno G.A. (2012). Coping Flexibility, Potentially Traumatic Life Events, and Resilience: A Prospective Study of College Student Adjustment. J. Soc. Clin. Psychol..

[39] Pereda N., Forns M., Kirchner T., Muñoz D. (2009). Use of the Kidcope to Identify Socio-economically Diverse Spanish School-age Children’s Stressors and Coping Strategies. Child.

[40] Glasscock D.J., Andersen J.H., Labriola M., Rasmussen K., Hansen C.D. (2013). Can Negative Life Events and Coping Style Help Explain Socioeconomic Differences in Perceived Stress among Adolescents? A Cross-Sectional Study Based on the West Jutland Cohort Study. BMC Public Health.

[41] Plieger T., Melchers M., Montag C., Meermann R., Reuter M. (2015). Life Stress as Potential Risk Factor for Depression and Burnout. Burn. Res..

[42] Montero-Marin J., Prado-Abril J., Piva Demarzo M.M., Gascon S., García-Campayo J. (2014). Coping with Stress and Types of Burnout: Explanatory Power of Different Coping Strategies. PLoS ONE.

[43] Horwitz A.G., Hill R.M., King C.A. (2011). Specific Coping Behaviors in Relation to Adolescent Depression and Suicidal Ideation. J. Adolesc..

[44] Li Y., Ye Y., Yang X., Huang J., He Z., Zhou X. (2025). Patterns in Transitions of Coping and Their Associations with Adolescents’ Post-Traumatic Distress and Growth: A Random Intercept Latent Transition Analysis. Anxiety Stress Coping.

[45] Spirito A., Stark L.J., Williams C. (1988). Development of a Brief Coping Checklist for Use with Pediatric Populations. J. Pediatr. Psychol..

[46] Vigna J.F., Hernandez B.C., Kelley M.L., Gresham F.M. (2010). Coping Behavior in Hurricane-Affected African American Youth: Psychometric Properties of the Kidcope. J. Black Psychol..

[47] Schwarzer R., Jerusalem M. (1995). Generalized Self-Efficacy Scale. Measures in Health Psychology: A User’s Portfolio. Causal and Control Beliefs.

[48] Cohen S., Kamarck T., Mermelstein R. (1983). Perceived Stress Scale. Measuring Stress: A Guide for Health and Social Scientists.

[49] Andresen E.M., Malmgren J.A., Carter W.B., Patrick D.L. (1994). Screening for Depression in Well Older Adults: Evaluation of a Short Form of the CES-D. Am. J. Prev. Med..

[50] Muthén L.K., Muthén B.O. (1998). Mplus User’s Guide.

[51] Collins L.M., Lanza S.T. (2010). Latent Class and Latent Transition Analysis: With Applications in the Social, Behavioral, and Health Sciences.

[52] Lo Y. (2001). Testing the Number of Components in a Normal Mixture. Biometrika.

[53] Nylund K.L., Asparouhov T., Muthén B.O. (2007). Deciding on the Number of Classes in Latent Class Analysis and Growth Mixture Modeling: A Monte Carlo Simulation Study. Struct. Equ. Model. A Multidiscip. J..

[54] Enders C.K. (2001). The Impact of Nonnormality on Full Information Maximum-Likelihood Estimation for Structural Equation Models with Missing Data. Psychol. Methods.

[55] Asparouhov T., Muthén B. (2014). Auxiliary Variables in Mixture Modeling: A 3-Step Approach Using Mplus. Struct. Equ. Model..

[56] Wadsworth M.E. (2015). Development of Maladaptive Coping: A Functional Adaptation to Chronic, Uncontrollable Stress. Child Dev. Perspect..

[57] Schäfer J.Ö., Naumann E., Holmes E.A., Tuschen-Caffier B., Samson A.C. (2017). Emotion Regulation Strategies in Depressive and Anxiety Symptoms in Youth: A Meta-Analytic Review. J. Youth Adolesc..

[58] Lau B., Hem E., Berg A.M., Ekeberg Ø., Torgersen S. (2006). Personality Types, Coping, and Stress in the Norwegian Police Service. Personal. Individ. Differ..

[59] Donaldson D., Prinstein M.J., Danovsky M., Spirito A. (2000). Patterns of Children’s Coping with Life Stress: Implications for Clinicians. Am. J. Orthopsychiatry.

